# Supplementary material for: Comparative multiomics analysis of cell physiological state after culture in a basket bioreactor
Source: Sci Rep. 2022 Nov 23;12:20161. doi: 10.1038/s41598-022-24687-4 (PMC9686226; doi:10.1038/s41598-022-24687-4)
Supplement: Supplementary file 1 — Supplementary Information 1. [file 41598_2022_24687_MOESM1_ESM.zip › raw data/Transcriptomic raw data/6.Enrichment/1.GO/TE1vsTC1/DOWN/TE1vsTC1.down.GOcc_DAG.pdf]

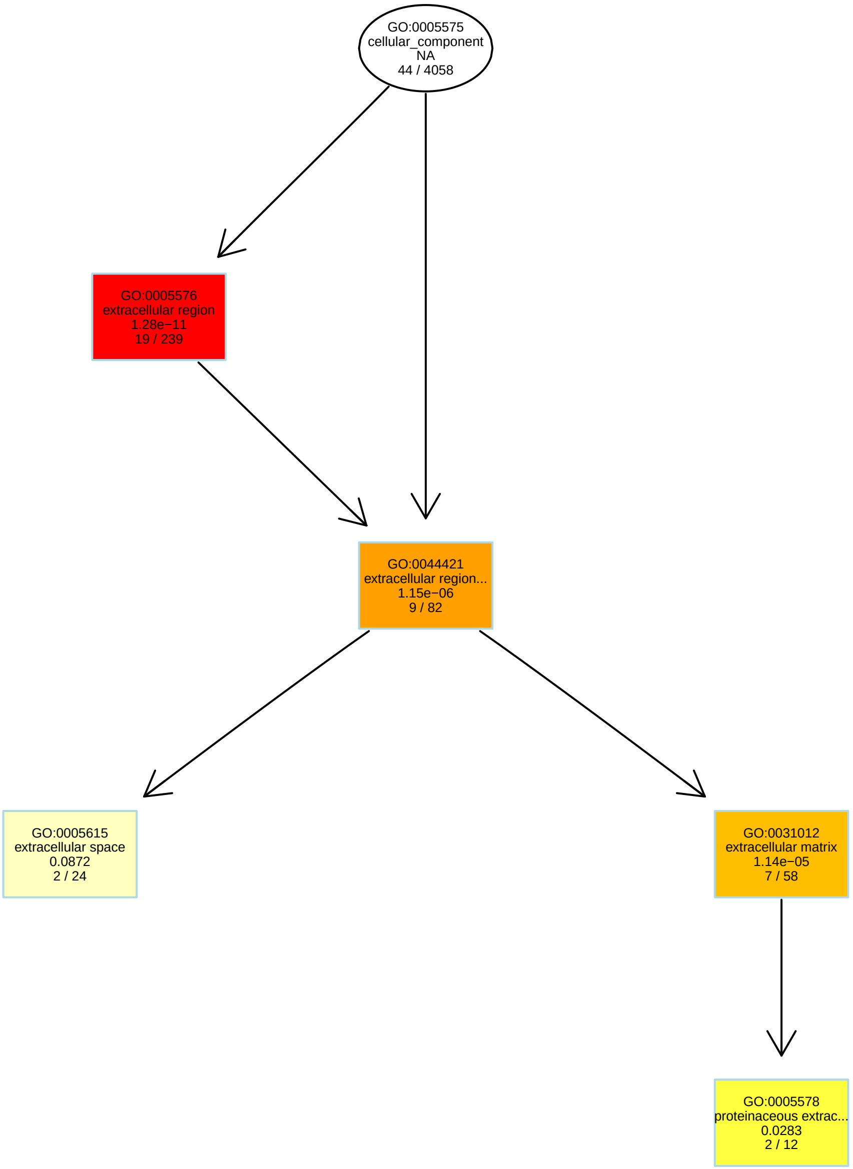
GO:0005575
cellular_component
NA
44 / 4058
GO:0005576
extracellular region
1.28e−11
19 / 239
GO:0044421
extracellular region...
1.15e−06
9 / 82
GO:0005615
extracellular space
0.0872
2 / 24
GO:0031012
extracellular matrix
1.14e−05
7 / 58
GO:0005578
proteinaceous extrac...
0.0283
2 / 12
